# From inertness to efficiency: immobilizing Au–Bi heterostructures on a graphite substrate to drive the hydrogen evolution reaction

**DOI:** 10.1039/d6ra08299h

**Published:** 2026-10-19

**Authors:** Kazi Hamidur Rashid, Nure Alam Siddique, Rehana Akter, Jahir Ahmed, M. Faisal, Jari S. Algethami, Farid A. Harraz, Jamal Uddin, Md Musfiqur Rahman, Nayan Ranjan Singha, Mohammad A. Hasnat

**Affiliations:** a Electrochemistry & Catalysis Research Laboratory (ECRL), Department of Chemistry, School of Physical Sciences, Shahjalal University of Science and Technology Sylhet-3114 Bangladesh mah-che@sust.edu; b Advanced Materials and Nano-Research Centre (AMNRC), Najran University Najran 11001 Saudi Arabia faharraz@nu.edu.sa; c Department of Chemistry, Faculty of Science and Arts, Najran University Najran 11001 Saudi Arabia; d Center for Nanotechnology, Department of Natural Sciences, Coppin State University 2500 W. North Ave. Baltimore MD 21216 USA; e Department of Mechanical Engineering, Iowa State University Ames Iowa 50011 USA; f Advanced Polymer Laboratory, Department of Polymer Science and Technology, Government College of Engineering and Leather Technology (Post-Graduate) Kolkata 700106 West Bengal India

## Abstract

In this study, we fabricated Au–Bi heterostructures on a pencil graphite (GP) support to generate hydrogen from water splitting under acidic conditions. Au particles, with their electronically filled 5d band, and Bi particles, known for their strong tendency toward surface oxide formation, exhibit limited intrinsic activity toward the HER. Remarkably, their integration produces an Au–Bi@GP electrode with substantially boosted hydrogen-evolution activity compared with the corresponding individual components. The Au@GP and Bi@GP electrodes require 486 mV and 442 mV to attain a 10 mA cm^−2^ current, respectively. But, while Au and Bi particles are integrated on the GP surface, the resultant Au–Bi@GP electrode requires only 139 mV to achieve the same current intensity showcasing its efficient hydrogen production. A Tafel slope of 141.4 mV dec^−1^ indicates that the HER is governed by the Volmer step indicating proton adsorption as the rate-limiting process. The electrode showed stable HER performance with negligible loss after 25 h of chronoamperometry test and 1000 voltametric runs. These results reveal an intriguing ‘inert-to-active’ transformation arising from the integration of two naturally sluggish HER (Au and Bi) elements and highlight interfacial engineering as an auspicious strategy for developing unconventional electrocatalysts for acidic hydrogen generation.

## Abstract 2

In this study, we fabricated Au–Bi heterostructures on a pencil graphite (GP) support to generate hydrogen from water splitting under acidic conditions.

## Graphical abstract

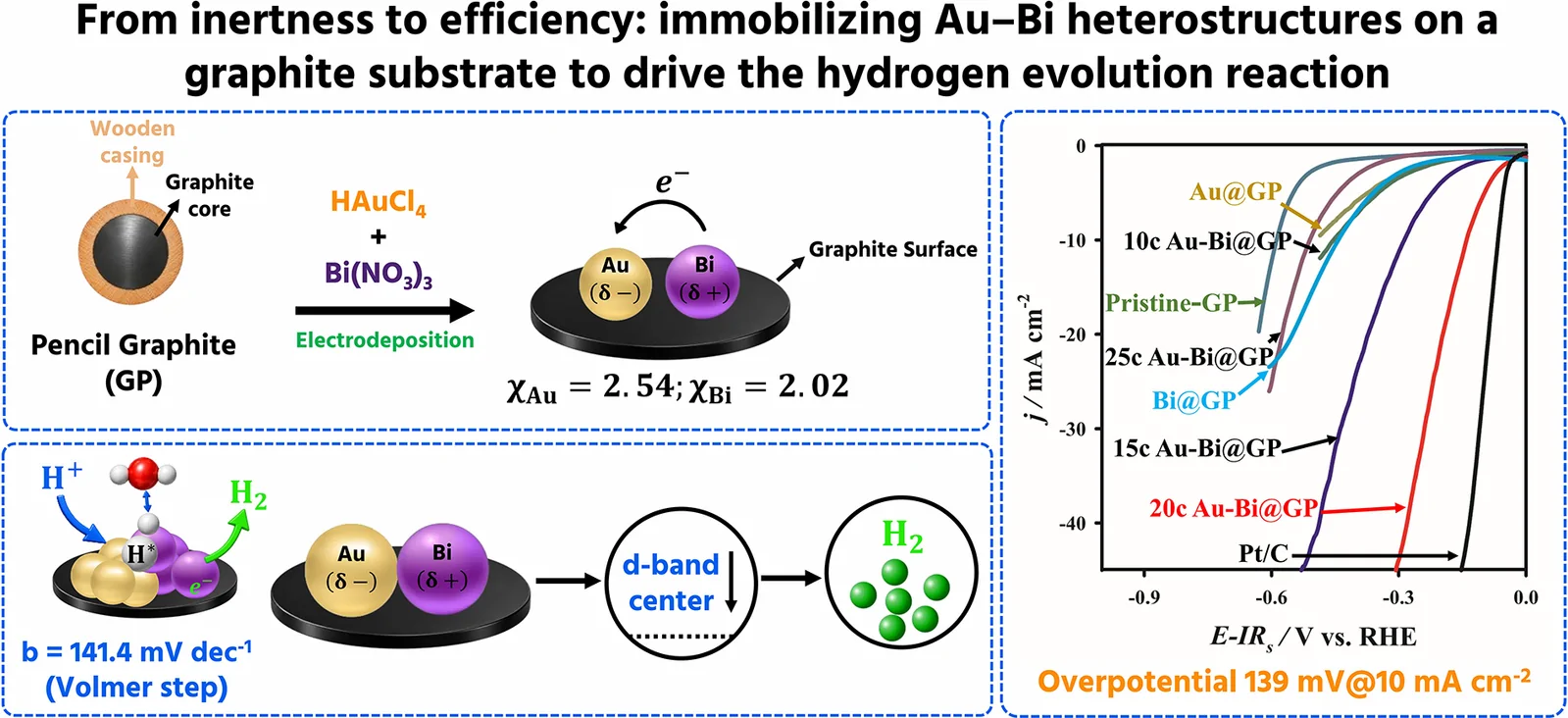

## 1 Introduction

In the last few decades, the rapid increase of global energy consumption, especially after the late 20th century, has become a critical challenge for sustainable development.^1,2^ Driven primarily by population growth, economic development, and rising living standards, global energy demand is projected to increase substantially, from approximately 16 TW in 2010 to 23 TW by 2030 and potentially 30 TW by 2050.^3–5^ Historically this increased demand has been met by fossil fuels such as coal, petroleum and natural gas which still provided around 80% of the global energy supply in 2016. This dependence has resulted in a global energy crisis. The crisis was compounded by geopolitical tensions that ripped through the gas and electricity markets in 2021 and 2022, sending prices soaring to record levels and threatening energy security on every continent.^3,6–8^ Aside from economic fluctuations, the environmental consequences of burning fossil fuels are dire. The huge CO_2_ emissions are the main cause of global warming and climate change while burning fossil fuels also produces harmful pollutants such as NO_*x*_ and SO_*x*_ which cause acid rain and contribute to millions of premature deaths each year.^9–12^ Nuclear energy is a low emission alternative but there are concerns about radioactive waste management and risks to safety.^13,14^

Thus, there is an urgent need to move towards sustainable energy systems in the world. Countries are increasingly adopting diversification policies based on renewable sources such as solar, wind, biomass and hydropower. Yet, because of the inherent intermittency and weather-dependence of solar and wind power means there is a significant need for advanced energy storage and conversion technologies to ensure grid stability.^15–19^ In this regard, hydrogen (H_2_) has become a game-changing energy carrier and a possible “Holy Grail” for the future carbon-free energy economy. Hydrogen is a colorless, transparent gas, with the highest gravimetric energy density of all molecular fuels (about several times higher than that of fossil fuels), and its combustion only produces water as a by-product, and thus a truly green energy cycle.^20–24^

However, more than 95% of the hydrogen production today is based on fossil resources *via* steam methane reforming, which uses finite fuels and emits considerable CO_2_.^25–28^ Alternative approaches such as biomass gasification, microbial electrolysis, thermochemical cycles and novel photocatalytic, piezocatalytic and pyrocatalytic routes are being explored but electrochemical water splitting remains the most feasible and efficient route for large scale high purity hydrogen generation, when powered by renewable electricity.^29–33^

In acidic media, HER starts with the Volmer step (H^+^ + e^−^ → H*), followed by either electrochemical desorption by the Heyrovsky step (H* + H^+^ + e^−^ → H_2_) or chemical desorption by the Tafel step (2H* → H_2_). The Tafel slope is an important measure of the rate determining step. Values around 120, 40 and 30 mV dec^−1^ correspond to Volmer, Heyrovsky and Tafel control respectively.^34–37^ The Sabatier principle states that the best catalyst binds hydrogen intermediates neither too strongly nor too weakly, with hydrogen adsorption free energy close to zero (Δ*G*_H*_ ≈ 0), minimizing the overpotential required to drive the reaction. This ideal is best approached by Pt and its alloys, which sit at the top of the volcano plot. However, their high cost, scarcity and limited long-term stability preclude large-scale deployment. Therefore, recent efforts have focused on decreasing the amount of noble metal while maintaining high activity. These include earth-abundant sulfides, carbides and phosphides and heterostructures in which a small amount of one component tunes the electronic properties of the others.^38–43^ The performance of any such catalyst is highly dependent on the choice of substrate. A good support is graphite, as the graphite pencil commercially available. It has high electrical conductivity, chemical stability in acidic electrolytes, low cost and easy availability. The surface also enables the direct growth of catalysts without polymeric binders that would otherwise block active sites and add resistance. Pristine graphite does not serve as a good HER catalyst, and its use is as a conductive scaffold for an active phase. Graphite electrodes coated with nickel, modified with cobalt, deposited with MoS_2_ and coated with Ni–Co have been studied, showing that graphite can support active layers, which improve electron transfer, increase the number of accessible active sites and enhance catalytic performance.^44–48^ Thus, a graphite substrate can be combined with a judiciously selected active phase to provide a low-cost, binder-free electrode.

A promising candidate for such a phase is bismuth. It is a low toxicity, earth abundant, “green” metal with a layered structure and readily accessible surface sites and has been proposed as a host for more active elements. However, its intrinsic HER performance is limited. Bi and its oxides bind hydrogen intermediates too strongly and have high overpotentials. This can be improved by alloying and doping, which change the electronic structure and expose more active sites. Bi, in contrast, offers an abundant, tunable and environmentally benign framework, but requires an electronic partner to bring its hydrogen adsorption closer to the optimum.^49–51^ Gold seems an unlikely partner. It is traditionally considered as catalytically inert due to its nobility and weak hydrogen adsorption. At the nanoscale however, Au behaves very differently and shows catalytic activity which is absent in the bulk form. When Au is alloyed with other metal, the electron structure can be redistributed and the interaction involving d-orbitals of Au can shift the free energy of hydrogen adsorption to the optimal value and facilitate the electron transfer. Au can also be added in small quantities, as small loadings of nanosized Au can change the electronic environment of the host material, with little contribution to the cost. This is the property that makes Au a suitable minor component on a Bi-based catalyst.^52–54^

The complementary limitations of Au and Bi suggest the existence of a synergy at their interface. Bi has abundant tunable surface sites but hydrogen binding is too weak, while Au has electronic modulation but few active sites when used at low loading. The electron density is expected to transfer from one metal to the other, polarizing the Au–Bi interface because of the electronegativity difference between the metals. Such redistribution should bring the hydrogen binding energy close to the thermoneutral situation and favor charge transfer in the Volmer step. The two metals are grown together on a conductive graphite scaffold, ensuring a short electron-transfer pathway between the catalytic interface and the current collector, and good interfacial contact without binder. We thus predict that a graphite-supported Au–Bi heterostructure will be superior to its single-metal counterparts and will achieve high activity at low noble-metal loading.^49,52,54–57^

However, there are few studies to investigate the enhancement of the HER performance of earth-abundant, Bi-based materials by trace amount of noble metal.^58–60^ Transition metal oxides and related abundant materials are stable and environmentally benign, but their low conductivity and slow kinetics make them intrinsically inactive.^61–64^ Au activation of a Bi host on a conductive carbon substrate through a binder-free electrochemical co-deposition strategy for acidic HER remains underexplored.^65^ To address this issue, the Au and Bi were electrochemically co-deposited on the surface of a graphite pencil to prepare a binder-free Au–Bi@GP heterostructure electrode and it's HER activity was evaluated in acidic electrolyte compared to bare graphite and the single-metal analogs. The design uses Au loading to make use of the synergistic interaction between the Bi host and the Au guest. The overpotential of the electrode is 139 mV and Tafel slope is 141.4 mV dec^−1^ with stable operation for 25 h. Its activity approaches that of commercial Pt/C, offering a durable, binder-free route that reduces the amount of noble metal required.

## 2 Materials and methods

### 2.1 Reagents and instrumentation

The chemicals used in this experiment were of analytical grade and selected for their remarkable purity. Gold(iii) chloride hydrate (HAuCl_4_·*n*H_2_O), bismuth(iii) nitrate pentahydrate (Bi(NO_3_)_3_·5H_2_O), nitric acid (HNO_3_) and sulfuric acid (H_2_SO_4_) were obtained from Sigma-Aldrich (St. Louis, MO, USA). To ensure reliable experimental results and to avoid contamination, aqueous solutions were produced in Milli-Q ultrapure water.

The electrochemical tests were carried out in a typical three-electrode set-up using two potentiostats CHI-660 (CHI Instruments, USA) and Auto-lab PGSTAT128N (Netherlands). The working electrode was a graphite electrode with a geometric surface area of 0.125 cm^2^ which was subjected to 20 cycles of Au and Bi co-deposition. The reference electrode was Ag/AgCl (3 M KCl) and the counter electrode was a platinum grid.

The structural and morphological properties of the synthesised materials were studied by Apreo 2S FESEM (Thermo Fisher Scientific, FEG, USA). The elemental composition was analyzed by energy-dispersive X-ray spectroscopy (EDX) using a TM3030Plus Miniscope (Hitachi Ltd). X-ray photoelectron spectroscopy (XPS) measurements were carried out using a delay-line detector (DLD) spectrometer (Thermo Fisher Scientific, SUST Central of Excellence) equipped with an Al Kα monochromatic X-ray source (1486.6 eV).

### 2.2 Electrochemical measurements

A standard PETRA PHOENIX-12B drawing pencil served to fabricate the electrode. First, it was fashioned into a lab-style electrode form, with the graphite core encased in biodegradable plastic. The graphite tip got wrapped and sealed in waterproof, eco-friendly plastic, leaving the working surface bare for reliable operation and peak efficiency. Prior to experiments, these graphite electrodes underwent through cleaning in nanopore water (17.3 MΩ cm) and 1 M H_2_SO_4_, then polishing on 120-grid sandpaper sheets. Next, the surface received a buff with a carbon pad, followed by a repeat rinse as noted earlier and air-drying at ambient temperature. Every water-based solution relied on nanopore water for preparation. Au and Bi solutions were combined in an 80 : 20 ratio (both at 0.05 M concentration). The Au–Bi mixture was then electrodeposited onto the graphite pencil (GP) surface *via* potential cycling from 0 to −1 V, repeated 20 times at a scan rate of 0.1 V s^−1^. Multiple such deposition cycles were performed on GP electrodes to optimize the cycling conditions.

All potentials reported here were recorded *versus* the Ag/AgCl (3 M KCl) reference electrode. Measurements were first taken relative to Ag/AgCl and subsequently converted to the reversible hydrogen electrode (RHE) scale using eqn (1),1
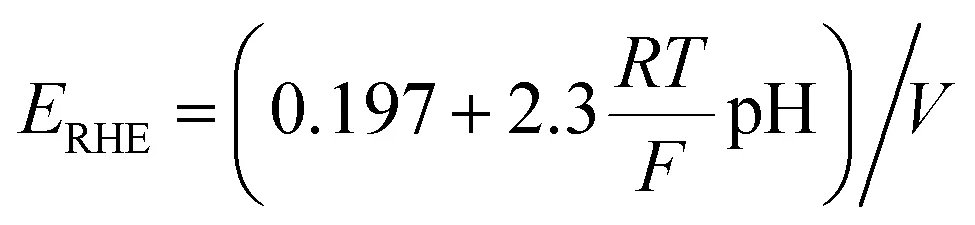


Electrochemical measurements utilized a CHI660E electrochemical workstation (CH Instruments, Inc., USA) and Autolab PGSTAT128N potentiostat (Metrohm Autolab, Netherlands). All charge and current densities were normalized to the geometric surface area of the graphite electrode substrate.

## 3 Results and discussion

### 3.1 Characterizations

The surface morphology of the modified electrodes snapped using scanning electron microscopy (SEM), is shown in Fig. 1(A). A fresh graphite surface is smooth, layered structure with typical axial grooves.^66^ However, after electrochemical co-deposition of Au and Bi particles, the surface morphology changes significantly. The Au–Bi@GP electrode exhibits rough, heterogeneous surface made up of irregular, cauliflower-shaped clumps scattered over the GP support.^67^ The tightly arranged nanosized clusters generate a porous network, which facilities the electrochemical activity of the surface. On the other hand, magnified SEM images reveals granular textures in the modified surface and also shows how nanoscale particles have fused into larger aggregates.^68^ Additional SEM images recorded at different magnifications and at different locations on the electrode (Fig. S1) show a similar nanoparticulate morphology over the whole surface, confirming the reproducibility of the deposit.^69^ This morphology effectively enhances HER by exposing active sites and the interconnected pores could improve charge transfer and gas release during HER.

**Fig. 1:**
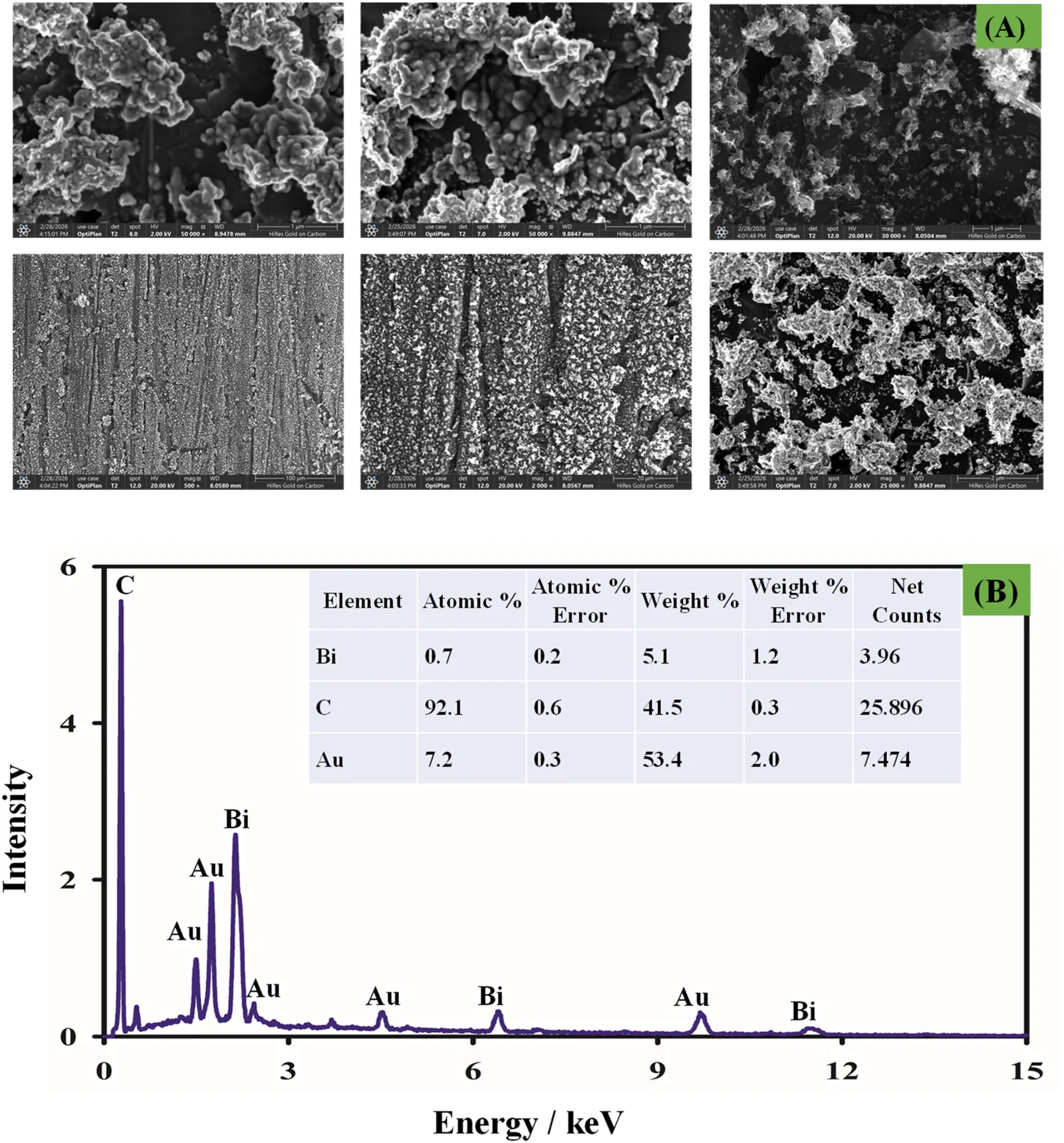
(A) SEM micrographs of 20c Au–Bi (prepared by 20 deposition cycles) modified GP surface at different magnifications, (B) EDX spectrum and corresponding elemental composition analysis confirming the presence of Au and Bi on the GP surface.

The elemental analysis of the Au–Bi@GP electrode using energy-dispersive X-ray spectroscopy (EDS). The EDS spectrum clearly shows carbon (C), gold (Au) and bismuth (Bi), which justifies that both metals are accurately loaded onto the GP support. The intense carbon signal comes from the graphite support, while the clear Au and Bi peaks confirm that they have been deposited there. Quantitative analysis reveals that carbon dominates the atomic percentage (92.1 at%) as expected from the graphite base. Gold exhibits 7.2 at% which proved its proper deposition. In contrast, Bi represents 0.7 at%, confirming its incorporation into the Au–Bi coating at a considerably lower concentration than Au.^70^ Despite of having lower percentage of Bi play an important role in electronic structure modification and catalytic behavior of Au.^71^ The absence of significant impurity peaks in the EDS spectrum indicates that the electrodeposition produces a high-purity composite material.^72^ Note that in case of Au–Bi@GP electrode Bi/Au ratio could be evaluated as 0.097. Hence, each AuBi_0.097_-unit cell has been considered as HER active site in this study.

X-ray photoelectron spectroscopy (XPS) was carried out to examine the surface composition and chemical states of the Au–Bi@GP electrode as shown in Fig. 2 and S2 in the SI.

**Fig. 2:**
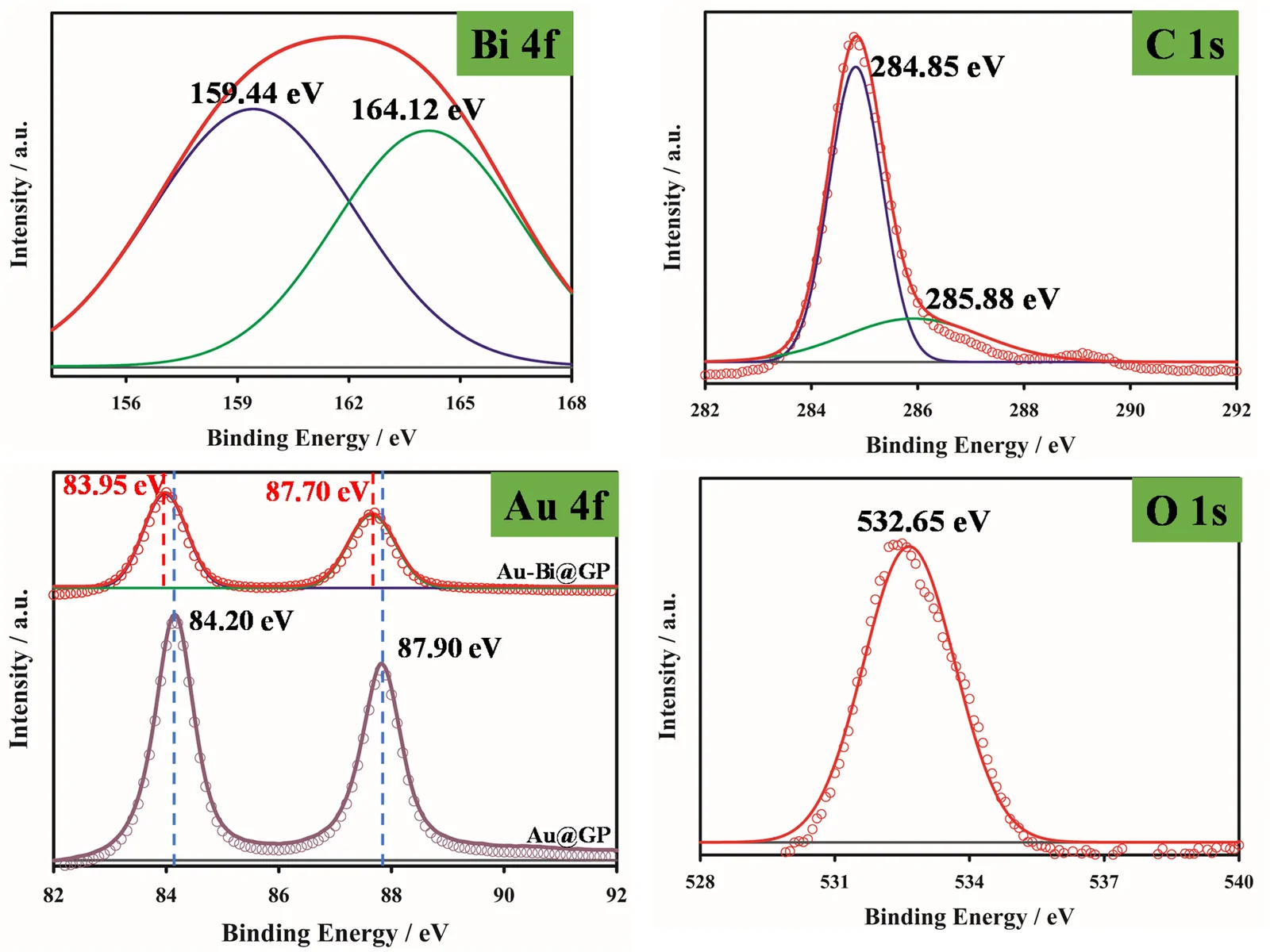
High-resolution deconvoluted XPS spectra of Bi 4f in 20c Au–Bi@GP, Au 4f (in Au@GP and 20c Au–Bi@GP), C 1s in 20c Au–Bi@GP, O 1s in 20c Au–Bi@GP, confirming the surface elemental composition and chemical states.

At first XPS spectrum for Bi particles in Au–Bi@GP was recorded and deconvoluted. The two peaks at 159.53 eV and 164.68 eV are ascribed to Bi 4f_7/2_ and Bi 4f_5/2_, respectively. These features are presumably attributed for oxidized bismuth Bi^3+^. Partial oxidation of Bi due to deposition or exposure to air may change the electronic structure of neighboring Au atoms.^73^ Therefore, XPS spectra for Au particles in Au@GP and Au–Bi@GP material were recorded next, where the spin–orbit-binding energy for 4f_7/2_ and Au 4f_5/2_ appeared clearly at 84.20 and 87.90 eV, respectively. Their positions and split confirmed the existence of Au particles mostly in Au^0^ state. Nothing jumps out for oxidation of gold; hence, the deposit stays stable on the GP substrate.^74^ To evaluate any peak binding energy shifting, XPS of Au particles in Au–Bi@GP surface were also recorded. In this instance, the Au 4f_7/2_ and Au 4f_5/2_ signals appeared at 83.95 and 87.70 eV, respectively. Therefore, it is evident that incorporation of Bi particles with Au@GP substrate caused a lowering of Au 4f_7/2_ and Au 4f_5/2_ binding-energy with an approximate value of 0.25 and 0.20 eV, respectively. This observation entails that while Bi particles were incorporated in Au@GP substrate, electron density on Au particles in the resultant Au–Bi@GP substrate increased.

Correspondingly, the C 1s spectrum, recorded for Au–Bi@GP, shows a pronounced peak at 284.86 eV from the sp^2^ carbon in graphite. There is also a minor one about 285.97 eV attributed to C–O bonds or defects, showing the graphite keeps its main structure but picks up certain groups that should help it play nicely with the electrolyte.^75^ Meanwhile, O 1s shows a peak about at 532.64 eV corresponding to metal–oxygen bonds and adsorbed OH or water.^76^

Overall, the XPS data backs up the bimetallic Au–Bi setup on the GP surface. Gold particles gains partially negative charges, while Bi is mostly oxidized. This combination of oxidation states, along with surface oxygen species, can tune the electrode's electronic properties and significantly improve HER activity.

The crystal structure of the Au–Bi@GP electrode was investigated by X-ray diffraction, as shown in Fig. 3. The diffraction pattern exhibits prominent peaks at approximately 2*θ* = 38.2°, 44.4°, 77.5°, and 81.7°, which can be indexed to the (111), (200), (311), and (222) planes, respectively, of face-centered cubic (fcc) metallic Au (JCPDS no. 04-0784). The particularly intense Au(111) reflection indicates a strong contribution from the (111) – oriented Au reflections are consistent with the formation of crystalline metallic Au during electrodeposition.^77^

**Fig. 3:**
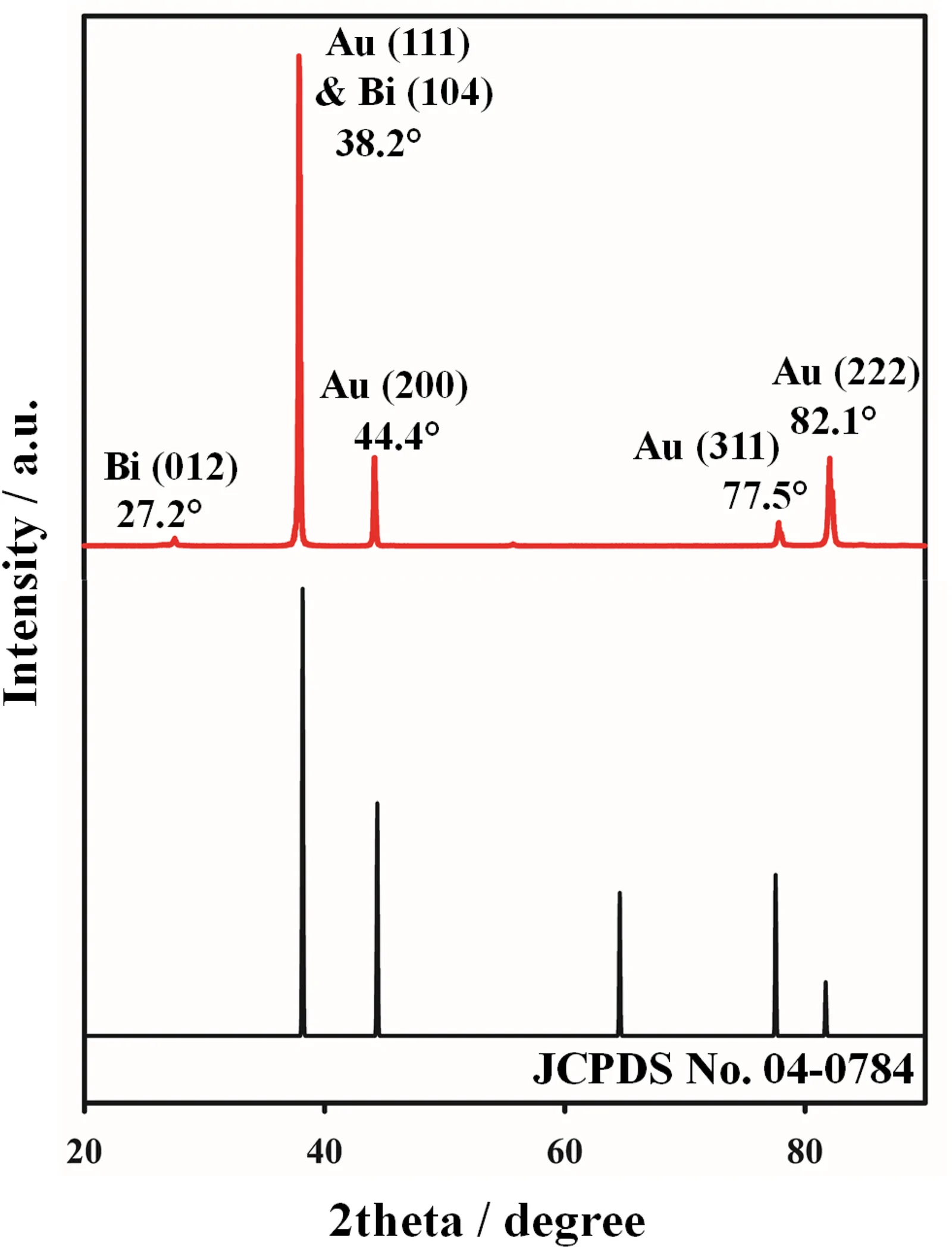
X-ray diffraction pattern of the Au–Bi@GP electrode showing the characteristic reflections of crystalline Au and Bi-containing phase.

A weak diffraction feature is also observed at approximately 27.2°, which may arise from the (012) reflection of rhombohedral metallic Bi. Metallic Bi commonly exhibits characteristic reflections around 27.2°, 37.9°, 39.6°, 48.7°, and 64.5°, corresponding to its rhombohedral crystal structure.^78^ In case of Au–Bi@GP pattern, however, the Bi-related reflections are comparatively weak and are partly overlapped by the intense Au reflections, particularly in the region around 38°–45°. Therefore, the XRD results support the presence of crystalline Au together with a Bi-containing phase, but the relatively low intensity of the Bi reflections suggests that the Bi component is either present in a comparatively low crystalline fraction or has a small crystallite size or dispersed nature. The absence of additional diffraction peaks attributable to an intermetallic Au–Bi phase, along with the distinct reflections assigned to metallic Au and Bi, indicates the coexistence of crystalline Au and Bi-containing phases as heterostructures.

### 3.2 HER performance

The open circuit potential (OCP) is a common primary indicator for evaluating the behavior of an electrode. It reflects the intrinsic potential of an electrode surface under equilibrium conditions in the absence of an applied current.^79^ In this study, OCP values of the prepared electrodes pristine-GP, Au@GP, Bi@GP and Au–Bi@GP were systematically estimated, as shown in Fig. 4(A, B) and Table 1. A pristine-GP demonstrated OCP at 0.62 V *vs.* RHE. When Bi particles were deposited onto the GP surface, the OCP value lifted to 0.89 V, which is consistent with literature.^80^ In contrast when Au is incorporated on the surface OCP shifted negatively and appeared at 0.58 V. However, when the Au and Bi particles were co-deposited onto the GP surface (20c Au–Bi@GP), the resultant surface exhibited the minimum OCP of 0.35 V. This negative shifting is the indication of electron enrichment on the electrode surface. This observation is thus infers the favorable environment for HER performance by tuning surface d bands promoting faster proton adsorption and enhanced HER kinetics.^81,82^

**Fig. 4:**
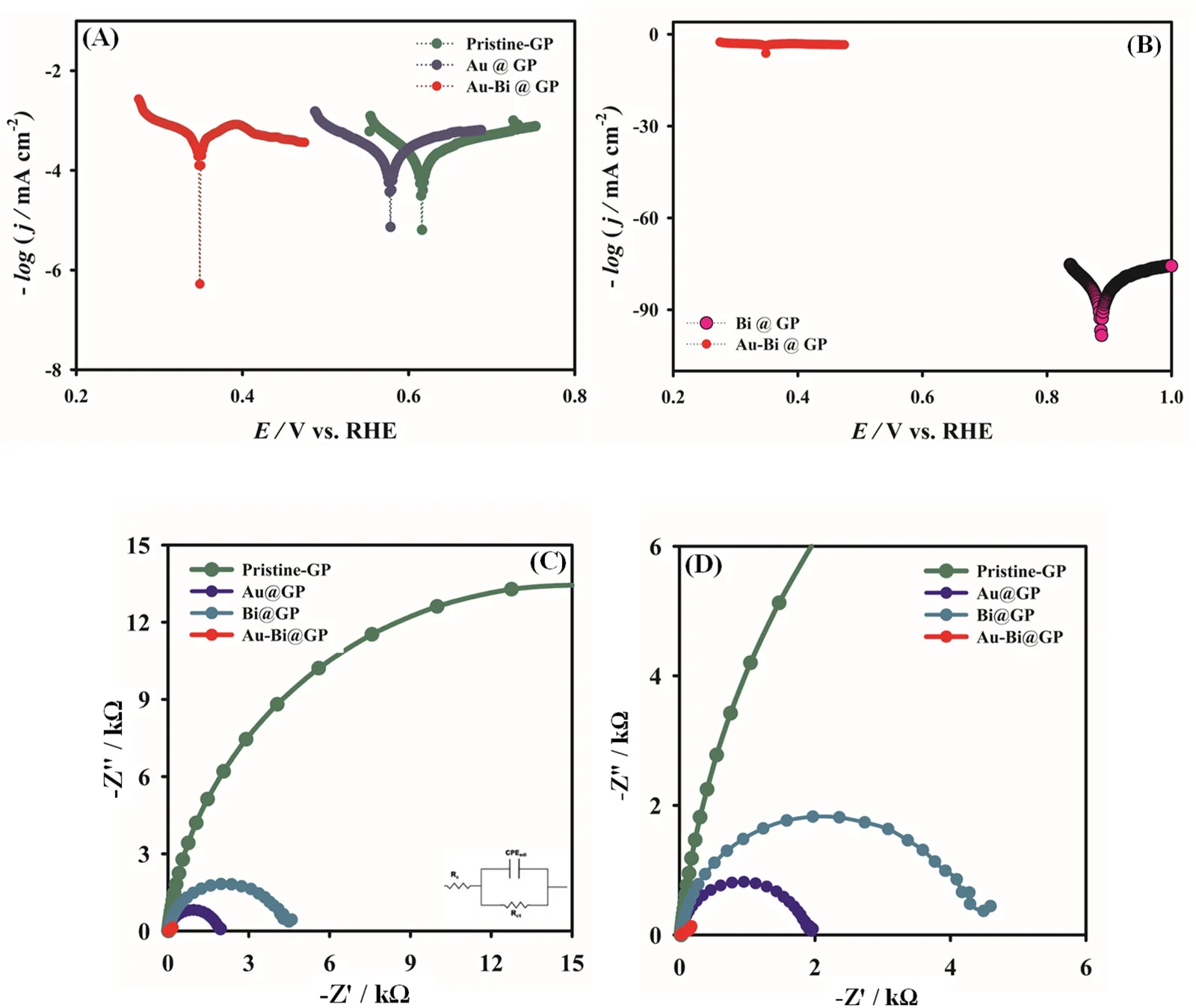
(A and B) Linear polarization curves of pristine-GP, Au@GP, Bi@GP and 20c Au–Bi@GP electrodes in 0.5 M H_2_SO_4_ with a scan rate of 0.1 V s^−1^. (C) Nyquist plots of the corresponding electrodes measured in 0.5 M H_2_SO_4_ at the open-circuit potential (OCP) to show the charge-transfer characteristics and HER performance of the electrodes, (D) Nyquist plots zoomed for high frequency region.

**Table 1:** Comparative HER kinetic parameters of pristine-GP, Bi@GP, Au@GP, and Au–Bi@GP electrodes

Electrodes	Pristine-GP	Au@GP	Bi@GP	Au–Bi@GP
OCP/V (RHE)	0.62	0.58	0.89	0.35
*E*_onset_/mV	479	173	220	39
*b*_T_/mV dec^−1^	177	379	261	141.4
*η*_10_/mV	590	486	442	139
TOF/s^−1^	—	0.24	0.13	0.37

To gain deeper insight into the interfacial charge-transfer dynamics at each electrode, electrochemical impedance measurements were performed.^83^ The Nyquist plots were obtained for the pristine-GP, Au@GP, Bi@GP and Au–Bi@GP electrodes at OCP potential are 0.337, 0.271, 0.621, and 0.059 V *vs.* Ag/AgCl (3 M KCl), respectively, shown in Fig. 4(C and D), and the corresponding equivalent circuit parameters derived from fitting using a Randles-type circuit (*R*_s_ + CPE/*R*_ct_) are summarized in Table S1. The solution resistance (*R*_s_) remained almost constant for all of pristine-GP, Au@GP, Bi@GP structures. The *R*_s_ of Au–Bi@GP electrode was slightly lower (20.7 Ω) which can be attributed to the enhanced electrical conductivity by the bimetallic interface.^84^ The pristine-GP electrode had the highest charge transfer resistance *R*_ct_ = 29.6 kΩ among all the tested electrodes, which infers slow interfacial charge transfer kinetics and reduced electrocatalytic activity towards the HER. The *R*_ct_ reduced to 4.27 kΩ after modification with bismuth nanostructures Bi@GP, suggesting that the charge-transfer capability of the electrode is improved partially after the deposition of Bi. The introduction of gold nanoparticles on GP electrode further reduced the *R*_ct_ to 1.85 kΩ, consistent with gold's well-known ability to accelerate electron-transfer processes.^85^

Notably, the Au–Bi@GP electrode exhibited lowest *R*_ct_ (0.024 kΩ), nearly three orders of magnitude lower than that of pristine-GP. This exceptional result demonstrates that the synergistic interaction between gold and bismuth at the electrode interface substantially facilitated the charge transfer, which is expected to translate directly into enhanced HER electrocatalytic performance. The significant drop in *R*_ct_ for Au–Bi@GP is further corroborated by the markedly compressed semicircle observed in its Nyquist plot.^86^ The constant phase element (CPE) values also varied across the electrodes, with Au–Bi@GP exhibiting the lowest CPE value is 2.43 and a notably reduced ideality factor *N* = 0.58, compared to values approaching unity for the other electrodes. The lower *N* value for Au–Bi@GP suggests a more heterogeneous and electrochemical active surface, consistent with the formation of a bimetallic nanostructured interface that introduces additional active sites and surface irregularities conducive to HER activity.^87,88^

To further study the possible dependence of the interfacial charge-transfer kinetics, EIS spectra were also recorded for all electrodes under different applied potentials (−0.297 to 0.003 V *vs.* RHE), and the combined Nyquist plots are presented in Fig. S3. The semicircle diameter, *R*_ct_, was highly dependent on the applied potential for all electrodes, which indicates the potential-dependent driving force for proton reduction. At the more negative potentials (−0.297 and −0.247 V *vs.* RHE) where the HER proceeds appreciably, Au–Bi@GP showed the smallest semicircle by far (*R*_ct_ ≈ 1–8 kΩ), while pristine-GP, Au@GP and Bi@GP showed much larger semicircles in the range of tens to hundreds of kΩ. This trend is consistent with the low *R*_ct_ obtained at OCP and confirms that the bimetallic Au–Bi interface promotes a faster charge transfer at HER-relevant conditions.

In this work, the HER activity of all tested electrodes such as pristine-GP, Bi@GP, Au@GP, and Au–Bi@GP was systematically examined in a 0.5 M H_2_SO_4_ electrolyte, with commercial Pt/C used as a benchmark catalyst (Fig. 5(A)). A pristine-GP shows very low HER catalytic activity, requiring a high overpotential of 590 mV to reach 10 mA cm^−2^, which is expected due to its limited number of active sites and sluggish electron transfer. Even after deposition of Au particles on GP surface, the resultant Au@GP electrode does not show improvement; instead, the overpotential slightly increased to 486 mV, suggesting that Au alone is not effective for HER on the GP substrate under the experimental condition. In contrast, the introduction of Bi significantly enhanced the performance. The Bi@GP electrode exhibited a much lower overpotential of 442 mV indicating that Bi particles can provide relatively more favorable environment for proton adsorption and hence could improve the reaction kinetics compared to the graphite and Au-modified electrodes. The most impressive HER activity was observed for the bimetallic Au–Bi@GP electrode, which delivered an exceptionally low overpotential of only 139 mV at 10 mA cm^−2^. This value is comparable to the commercial Pt/C catalyst (66 mV), highlighting the strong synergistic effect between Au and Bi. This synergy likely modifies the electronic structure of the surface, facilitating faster charge transfer and optimizing hydrogen adsorption during HER.

**Fig. 5:**
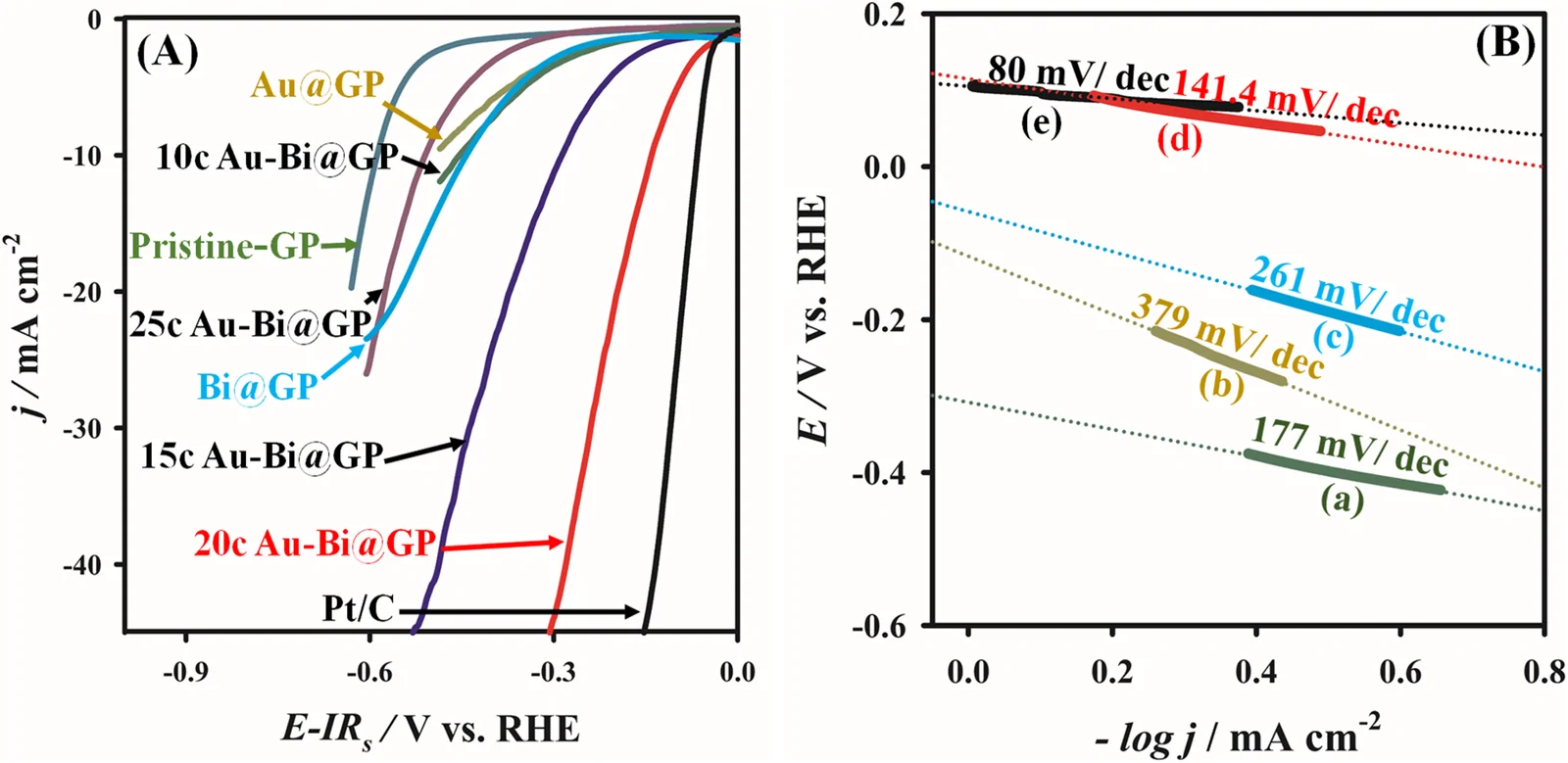
(A) Linear sweep voltammetry (LSV) curves of pristine-GP, Au@GP, Bi@GP, Au–Bi@GP (10c, 15c, 20c, 25c) and Pt/C electrodes, (B) Tafel plots of all electrodes according to (A).

Next, effect of Au–Bi content on the HER performance was investigated. For this purpose, a variable amount of Au–Bi was deposited on GP substrate by varying number of deposition cycles. It is evident from the polarization curves that the catalytic performance strongly depends on the number of deposition cycles, hence Bi–Au content. An electrode (10c Au–Bi@GP) prepared by 10 deposition cycles shows relatively decent HER activity suggesting that the preliminary formation of the bimetallic layer creates active sites for hydrogen evolution. However, the current response is still limited, indicating that the surface coverage may not be sufficient at this stage. As the Au–Bi deposition cycle number was 15–20, a noticeable further improvement in HER performance is observed. In particular, the 20c Au–Bi@GP electrode exhibited the best activity among all the fabricated electrodes delivering higher current density at less negative potentials. This observation indicates that an optimal amount of Au–Bi was deposited on GP surface with 20 cycles providing a balanced surface with abundant active sites and efficient charge transfer pathways. The improved performance at this stage can be linked to better surface uniformity and stronger interaction between Au and Bi.

Interestingly, further increasing the cycle number to 25c led to a decline in HER performance. Although more material was deposited, the HER activity became poorer compared to the optimized 20c Au–Bi@GP electrode. This can be attributed to excessive loading of Au–Bi particles might have caused particle aggregation or the formation of a thicker layer that blocked the active sites and inhibited electron transport.

To unveil the electrokinetics of HER at the fabricated electrodes and identify the mechanistic pathway controlling the hydrogen evolution reaction, Tafel analyses were conducted for the Au–Bi@GP, Bi@GP, Au@GP, and pristine-GP electrodes based on the polarization curves shown in Fig. 5(B), with commercial Pt/C included as a reference catalyst.^89^ The Tafel slope (*b*_T_) was determined from eqn (2),^90^ where *E*° represents the equilibrium potential of the electrode, *E* is the applied potential referenced against the reversible hydrogen electrode (RHE), *j*_0_ is the exchange current density, and *b*_T_ is the Tafel slope.

2 log(*j*) = log(*j*_0_) + *b*_T_*η*

Here,*η* = *E* − *E*°

It is widely accepted that electrochemical reactions involving hydrogen evolution proceed through sequential multi-electron transfer steps. In an acidic electrolyte, HER advances through the mechanistic sequence outlined with eqn (3)–(5), where specific step potentially acts as the rate-determining step (RDS):

3 H_3_O_aq_^+^ + e^−^ → H_ad_

4 H_ad_ + H_3_O_aq_^+^ + e^−^ → H_2_(g) + H_2_O(l)

5 H_ad_ + H_ad_ → H_2_(g)

The HER is initiates by the electrochemical discharge of protons from the electrolyte to produce adsorbed hydrogen atoms (H*), which is an elementary step called the Volmer step (eqn (3)). In the following Heyrovsky step (eqn (4)), the adsorbed hydrogen reacts with a proton from the electrolyte and an electron to form molecular hydrogen (H_2_). Alternatively, two adsorbed hydrogen atoms may combine directly to form H_2_ on the electrode surface widely known as Tafel step (eqn (5)).

The Tafel slope (*b*_T_) provides mechanistic insights into the hydrogen evolution reaction (HER). With a charge-transfer coefficient (*α*) of 0.5 for the rate-determining step (RDS), *b*_T_ can be expressed as a function of the number of electrons (*n*) transferred prior to the RDS. When no preceding electron transfer occurs (*n* = 0), a slope close to 120 mV dec^−1^ is anticipated, pointing to the Volmer step as the bottleneck step. For *n* = 1, a slope of around 40 mV dec^−1^ is observed, which agrees with a Heyrovsky-limiting route, indicating that one electron is transferred before the RDS. For a completely chemical RDS (*n* = 2) with two electrons preceding it, as in the Tafel recombination stage, the anticipated slope is approaching 30 mV dec^−1^.^91^ In the present work, the pristine-GP electrode exhibited the poorest catalytic activity among all the examined electrodes. This was verified by its unusually high Tafel slope of 177 mV dec^−1^. This value shows very slow reaction kinetics, in agreement with the absence of any electro catalytically active species on the graphite surface. For the Au@GP electrode, the Tafel slope remained significantly high at 379 mV dec^−1^ suggesting that Au deposition alone at this loading could not accelerate the HER process (see Table 1). The poor performance of Au catalyst is attributed due to Au's completely filled 5d band lies well below the Fermi level compared to Pt-group metals, giving it poor orbital overlap with the H 1s orbital and correspondingly weak chemisorption of reaction intermediates.^92^ Meanwhile, Bi@GP displayed a Tafel slope of 261 mV dec^−1^, which is a moderate improvement over pristine-GP electrode. But still this electrode is sufficiently sluggish to exhibit acceptable HER activity. Note that Bi is highly unstable metal in exposure to air and its particles are readily converted into Bi_2_O_3_ which intrinsically generates positive charges (also proven by the most positive OCP as per Fig. 4(A and B)) on its surface,^93^ hence the Bi@GP might have shown weak to moderate affinity towards incipient H intermediates. Thus, its lower intrinsic binding affinity for hydrogen limits its ability to catalyse the HER at a significant rate.^94^

Interestingly, the bimetallic 20c Au–Bi@GP electrode exhibited a notable improvement in HER kinetics (see Fig. S4) with a significantly lower Tafel slope of 141.4 mV dec^−1^. This number is within the range generally attributed to a Volmer-limited process, where the entire reaction rate is controlled by the first electrochemical proton discharge step. The dramatic lowering of the Tafel slope as compared to Au@GP or Bi@GP alone strongly indicates a synergistic interaction between Au and Bi on the GP surface.

It is well known that Au metal is more electronegative than that of Bi metal (*χ*_Au_ = 2.54 > *χ*_Bi_ = 2.02). Consequently, while Bi and Au particles were combined, at the resultant Au–Bi interfaces, charge transfer occurs from Bi to Au, generating slightly electron-rich Au (Au^*δ*−^) sites and electron-deficient Bi (Bi^*δ*+^) sites which is also convinced from XPS analysis. This redistribution of electron density shifts the d-band/surface electronic structure of both metals, moving free energy (Δ*G*_H*_) closer to thermoneutral on at least one component—increasing the rate of H* adsorption/desorption.

In this bimetallic configuration, Au is believed to enable quicker electron transport and provide energetically favourable locations for proton adsorption, while Bi modulates the local electronic structure around the active centers, thereby reducing the activation barrier for the Volmer step.^95^ This complementary effects of the components suggests that Au–Bi@GP electrode, fabricated by co-deposition method, may enhance electrocatalytic HER activity.^96^ For comparison, the commercial Pt/C electrode presented a Tafel slope of 80 mV dec^−1^ under identical conditions, which is in the predicted range for a Volmer–Heyrovsky pathway and acts as a benchmark.^97^ Although the Au–Bi@GP electrode has not reached this performance benchmark yet, the gap is much smaller than that of the individual component electrodes, indicating the promise of the bimetallic method. The research reveals that the Au–Bi@GP electrode is a substantial step towards the development of noble-metal reduced alternatives for practical H_2_ production applications.

### 3.3 TOF analysis

The turnover frequency (TOF) is one of the most important parameters for the performance evaluation of an electrocatalyst in facilitating a reaction. It is the number of mol of product formed per mol of active sites per unit time that reflects the intrinsic catalytic activity of the material. The higher the TOF value, the higher is the catalytic activity, which is an important benchmark for the performance evaluation and comparison of electrocatalytic materials. Thus, to evaluate intrinsic HER activity of the prepared electrode materials, turnover frequency (TOF) was determined from the electrochemically active surface area (ECSA)^98^ normalized LSV data, particularly from the 10 mA cm^−2^ current density. Rather than relying on geometric surface area alone, ECSA provides a more accurate basis for comparing different catalytic materials, since it reflects the actual interfacial area where electrochemical reactions can take place-making it indispensable for evaluating genuine catalytic activity.^99^ One of the most commonly used methods to estimate ECSA relies on measuring the double-layer capacitance (*C*_dl_), which captures the non-faradic current behavior observed within a potential window that is free of any redox processes.^100^ The relationship linking capacitive current (*i*) to the potential scan rate *ν*(=d*V*/d*t*) is mathematically described in eqn (6).6
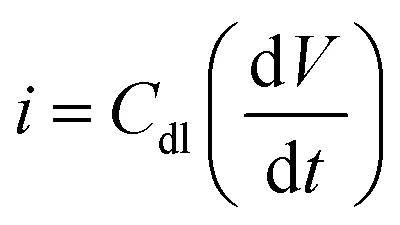


To estimate the ECSA of pristine-GP, Au@GP, Bi@GP, and Au–Bi@GP, measurements were carried out in 0.5 M H_2_SO_4_ solution using the aforementioned approach. CV data collected across a range of scan rates were used to plot the capacitive current response as a function of scan rate, as shown in Fig. S5. The slope derived from each of these plots yielded the corresponding *C*_dl_ values of 0.004, 0.026, 0.018, and 0.030 mF cm^−2^ for the electrodes pristine-GP, Au@GP, Bi@GP, and Au–Bi@GP, respectively. The electrochemically active surface area (ECSA) of all modified electrodes was then estimated using eqn (7),^101^ based on the known geometric area of the pristine-GP electrode (0.125 cm^2^).^102^7
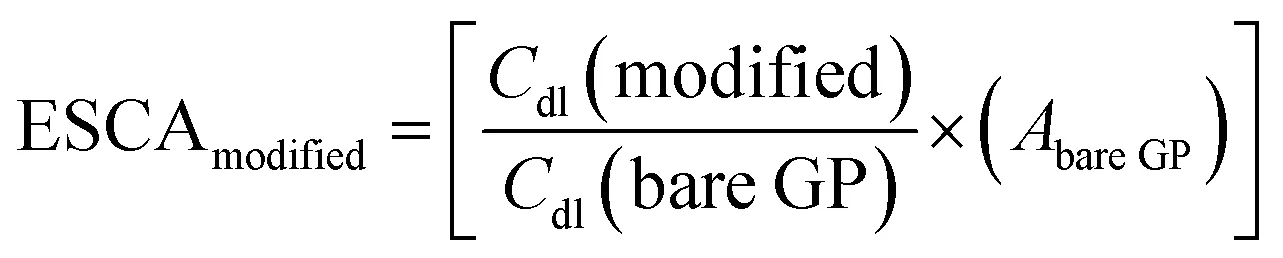


Using this equation ECSA was determined for GP, Au@GP, Bi@GP electrodes as 0.13, 0.81, 0.56 cm^2^, respectively. When Au–Bi was co-deposited over GP using 20 deposition cycles, the ECSA reached 0.94 cm^2^, reflecting a favorable equilibrium between surface coverage and the accessibility of active sites within the bimetallic composite. This improved reactive area, which is constantly higher than those obtained for the single-metal modified electrodes, points forth the suitability of Au–Bi@GP as a candidate for the HER.^103^ The ECSA normalized LSV curves are then shown in Fig. S6 in the SI.

The TOF values in this work were then evaluated using the expression given in eqn (8).^101,104^8
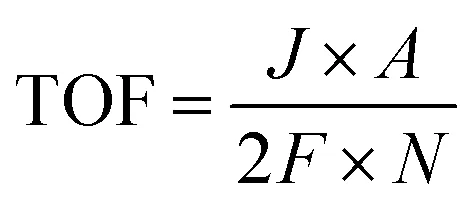
Here, *J* = 10 × 10^−3^ ampere cm^−2^, *A* = ECSA, *F* is Faraday constant and *N* is the number of moles of metal atoms employed for obtaining HER activity. In case of Au@GP electrode, *N* was calculated by integrating the area under deposition CV cycles. In case of Bi@GP electrode, after voltammetric deposition of Bi particles on GP surface, number of moles of Bi was determined by anodic striping voltammetry (see Fig. S7). Meanwhile, in case of Au–Bi@GP electrode, after fabricating the electrode, the moles of the Bi particles were determined using anodic striping voltammetry. Then, number of moles of Au particles were determined from the ratio of Bi/Au particles as observed by the EDS analysis, where *N* was the moles of AuBi_0.097_ unit cell considered for HER activity. However, because the number of catalytically active sites on a pure graphite surface could not be experimentally determined, hence, a rigorous site-normalized TOF could not be assigned to the bare graphite electrode. The TOF values for fabricated electrodes were evaluated as 0.13, 0.24, and 0.37 s^−1^ for Bi@GP, Au@GP, and Au–Bi@GP respectively, reflecting the relatively modest intrinsic catalytic activity of the developed electrodes. This observation suggest that among the three developed electrodes Au–Bi@GP electrode exhibited the most intrinsic activity pertaining to hydrogen evolution reaction under the experimental condition. Consequently, the above discussion entails that while two inert metals (Au and Bi) are combined over the GP scaffold, the resulting electrode not only apparently enhances the HER activity but also it intrinsically promote noble active sites to accelerate HER activity *via* redistribution of electronic properties of the participating metal atoms.

### 3.4 Stability

The electrochemical durability of the 20c Au–Bi@GP electrode was evaluated through two complementary methods: accelerated CV cycling and chronoamperometric (CA) testing which are illustrated in Fig. 6(A and B), respectively.

**Fig. 6:**
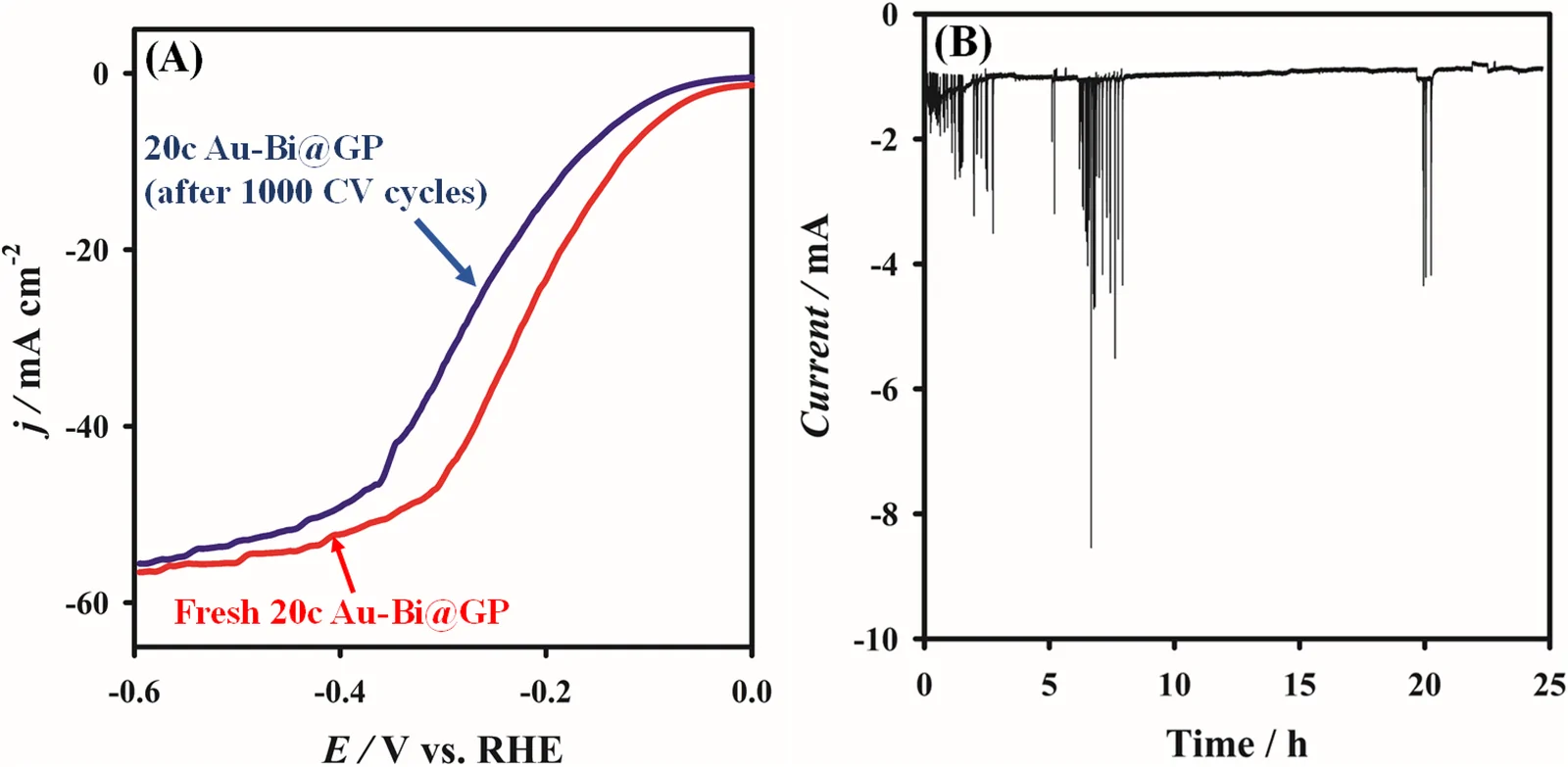
Stability evaluation of the 20c Au–Bi@GP electrode toward HER performance: (A) polarization curves recorded before and after 1000 continuous CV cycles, (B) chronoamperometric response showing the long-term operational stability over extended electrolysis time.

As evident from Fig. 6(A), the overall cathodic current–potential profile is largely preserved after cycling, confirming that the bimetallic Au–Bi active phase anchored on the GP surface does not undergo irreversible structural breakdown. A moderate reduction in current density is observed in the post-cycling curve, particularly in the −0.105 to −0.35 V *vs.* RHE region, which is consistent with minor surface reorganization of the bimetallic interface under repeated potential scanning rather than catastrophic deactivation. Repeated CV cycling simulates electrocatalyst stability under dynamic conditions.^90^ The fact is especially relevant when the electrode is exposed to intermittent electrochemical inputs. The retention of the general wave shape and the absence of any significant positive shift in onset potential confirm that the active Bi-containing phase does not dissolve or restructure irreversibly during the stress test.^105^ A potential limitation is that the electrical resistance of the graphite substrate may contribute to ohmic losses, particularly at high current densities. Further optimization of the substrate thickness and conductivity could therefore improve the high-current-density HER performance.

A continuous 25-hour chronoamperometric (CA) stability test was performed at a fixed applied potential. As shown in Fig. 6(B), the current rapidly stabilized near −1.0 mA and remains at this level throughout the test.^106^ During the subsequent 25-hour electrolysis, several transient current perturbations are observed, particularly around 1 h, 6–8 h, and 20 h. These short-lived fluctuations can be attributed mainly to the periodic formation, accumulation, and detachment of hydrogen gas bubbles from the electrode surface, which temporarily alters the affective electrode–electrolyte contract area. Importantly, after each perturbation, the current returns toward its established baseline without a continuous downward drift over the entire 25-hour period. The nearly constant baseline current throughout the prolonged electrolysis demonstrates the good operational stability of the electrode and indicates that the catalyst remains electrochemically active under continuous HER conditions.^107,108^

### 3.5 Post stability XPS

To probe the chemical state changes of each constituent element after HER operation, XPS was employed to provide a more detailed picture of the surface chemistry Fig. 7. The Au 4f spectrum still exhibits its typical spin–orbit doublet with binding energies of 84.00 eV (Au 4f_7/2_) and 87.70 eV (Au 4f_5/2_), which are consistent with metallic gold (Au^0^).^109^ The retention of these peak positions after long-term electrochemical testing confirms the excellent chemical stability of Au under HER conditions and confirms its role as a robust and continuously active site for hydrogen evolution. No evidence for a shift to higher binding energies was obtained, excluding a significant surface oxidation of Au during operation.

**Fig. 7:**
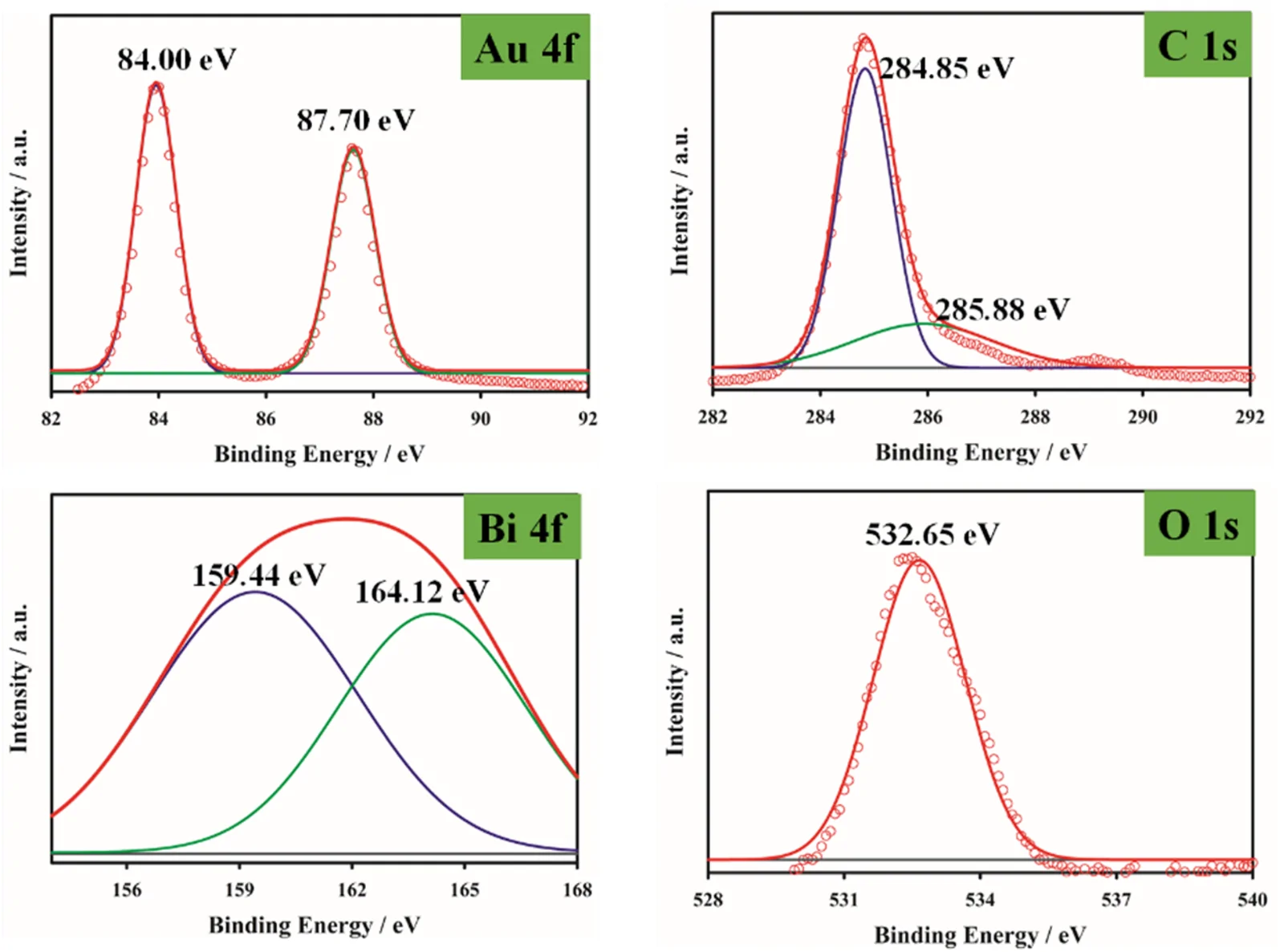
Post-electrolysis XPS analysis of the Au–Bi@GP electrode after prolonged HER operation.

The C 1s spectrum is basically unchanged after HER, with a strong peak centered at ∼284.85 eV, attributed to sp^2^-hybridized graphitic carbon, confirming the structural integrity of the pencil graphite support.^110^ A small shoulder at ∼285.88 eV still remains, which can be assigned to carbon associated with defects or residual oxygen containing functional groups (C–O species). The stability of the graphite framework during electrochemical testing confirms its suitability as a durable and conductive substrate for bimetallic electrocatalyst deposition.

The Bi 4f spectrum shows two peaks at approximately 159.44 eV and 164.12 eV, which are assigned to the Bi 4f_7/2_ and Bi 4f_5/2_ of oxidized Bi species (Bi^3+^).^111^ Comparing with data before characterization shows that Bi remains in its oxidized state after HER, but minor changes in relative peak intensities might be indicative of minor surface restructuring or partial Bi redistribution during operation. The oxidized Bi plays a critical role since the presence of Bi^3+^ species is known to influence the electronic environment of neighboring Au atoms through ligand-type interactions, leading to an enhancement in the proton adsorption and stabilization of reaction intermediates on the electrode surface.^112^ This electronic co-operation of Bi is in line with the synergistic HER activity observed in the Au–Bi bimetallic system.

The O 1s spectrum shows a peak at around 532.65 eV which is attributed to surface oxygen species such as metal–oxygen bonds and adsorbed hydroxyl species.^113^ The continued existence and possibly slight increase of these oxygen species after HER operation may be related to local surface oxidation processes and increased wettability of the electrode–electrolyte interface that can be beneficial for catalytic performance.

Overall, the post-characterization results suggest that the Au–Bi@GP electrode largely keeps its structural integrity and functional surface composition even though some morphological coarsening and minor compositional redistribution occurs during HER operation.

## 4 Conclusions

We have successfully produced a modified graphite electrode covered with a thin Au–Bi layer using a straightforward electrodeposition approach in the pursuit of developing cost-effective and efficient electrocatalysts for the hydrogen evolution reaction (HER) in acidic conditions. The obtained Au–Bi@GP electrode had extraordinary catalytic activity towards HER with a remarkably decreased overpotential and a smaller Tafel slope compared to the pristine-GP electrode, indicating the synergistic effect of Au and Bi in improving electrocatalytic activity. The Bi inclusion into Au matrix was critical to adjust the electronic structure of active sites, which would favour the hydrogen adsorption and desorption kinetics. In addition, the electrode showed good long-term electrochemical stability in acid medium, maintaining its activity during long term operation without any evident degradation. The electrochemical process gives us pure hydrogen, which is a very promising way of producing energy responsibly, without any carbon emissions. These results demonstrate a promising potential of the Au–Bi@GP electrode as a realistic and feasible alternative to typical Pt/C-based catalysis for HER applications.

## Conflicts of interest

There are no conflicts to declare.

## Supplementary Material

RA-016-D6RA08299H-s001

## Data Availability

The data supporting the findings of this study are provided within the article and its supplementary information (SI). Further information and supporting data are available from the corresponding author upon reasonable request. Supplementary information is available. See DOI: https://doi.org/10.1039/d6ra08299h.
