# Supplementary material for: From inertness to efficiency: immobilizing Au–Bi heterostructures on a graphite substrate to drive the hydrogen evolution reaction
Source: RSC Adv. 2026 Oct 19;16(48):64405–19. doi: 10.1039/d6ra08299h (PMC13679147; doi:10.1039/d6ra08299h)
Supplement: Supplementary file 1 [file RA-016-D6RA08299H-s001.pdf]

Supplementary Information (SI) for RSC Advances.
This journal is © The Royal Society of Chemistry

Supporting Information

From Inertness to Efficiency: Synergistic Au-Bi Heterostructures on Graphite Substrate to drive Hydrogen Evolution Reaction

Kazi Hamidur Rashid [a], Nure Alam Siddique [a], Rehana Akter [a], Jahir Ahmed[b,c], M. Faisal[b,c], Jari S. Algethami[b,c], Farid A. Harraz[b,c,*], Jamal Uddin [d], Md Musfiqur Rahman [e], Nayan Ranjan Singha [f], Mohammad A. Hasnat [a, *]

[a] *Electrochemistry & Catalysis Research Laboratory (ECRL), Department of Chemistry, School of Physical Sciences, Shahjalal University of Science and Technology, Sylhet-3114, Bangladesh*

[b] *Advanced Materials and Nano-Research Centre (AMNRC), Najran University, Najran 11001 Saudi Arabia*

[c] *Department of Chemistry, Faculty of Science and Arts, Najran University, Najran 11001 Saudi Arabia*

[d] *Center for Nanotechnology, Department of Natural Sciences, Coppin State University, 2500 W. North Ave., Baltimore, MD 21216, USA*

[e] *Department of Mechanical Engineering, Iowa State University, Ames, Iowa 50011, United States*

[f] *Advanced Polymer Laboratory, Department of Polymer Science and Technology, Government College of Engineering and Leather Technology (Post-Graduate), Kolkata 700106, West Bengal, India*

Corresponding authors: Mohammad A. Hasnat, Farid. A. Harraz

Correspondence: faharraz@nu.edu.sa (F.A.H) and mah-che@sust.edu (M.A.H.)

Table S1. EIS analysis of all tested electrodes in 0.5 M H_2SO_4

Electrode	R_s / Ω	R_{ct} / *k*Ω	CPE/$\square^{-1} s^{N}$	**N**
Bare-GP	23.2	29.6	20.7	0.946
Au@GP	23.2	1.85	20.9	0.946
Bi@GP	23.2	4.27	24.8	0.933
Au-Bi@GP	20.7	0.024	2.43	0.583

SEM images of Au-Bi@GP electrode were taken at different magnifications and different locations on the surface (Fig. S1) to give more morphological evidence for the Au-Bi deposit on the graphite substrate. In all the images the GP surface is covered densely and quite uniformly with nanosized quasi-spherical particles, with some larger clusters due to the aggregation of neighboring particles. As shown in the high magnification images (Fig. S1), the deposit is composed of closely packed nanoparticles with voids in between the particles, which gives the deposit a porous and rough surface. This morphology is reproducible in the different areas studied.

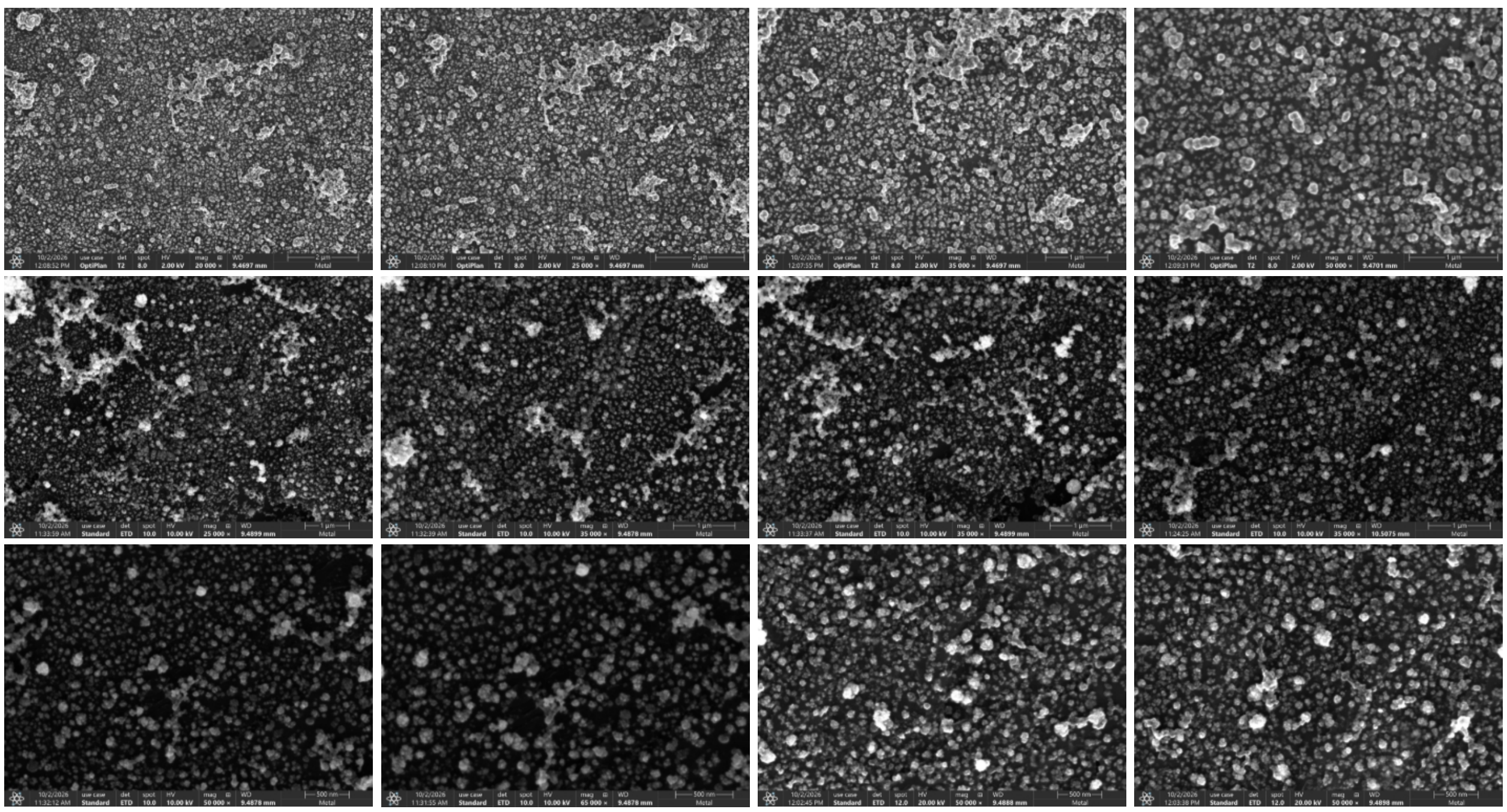

Figure S1. SEM images of the Au-Bi@GP electrode recorded at various magnifications.

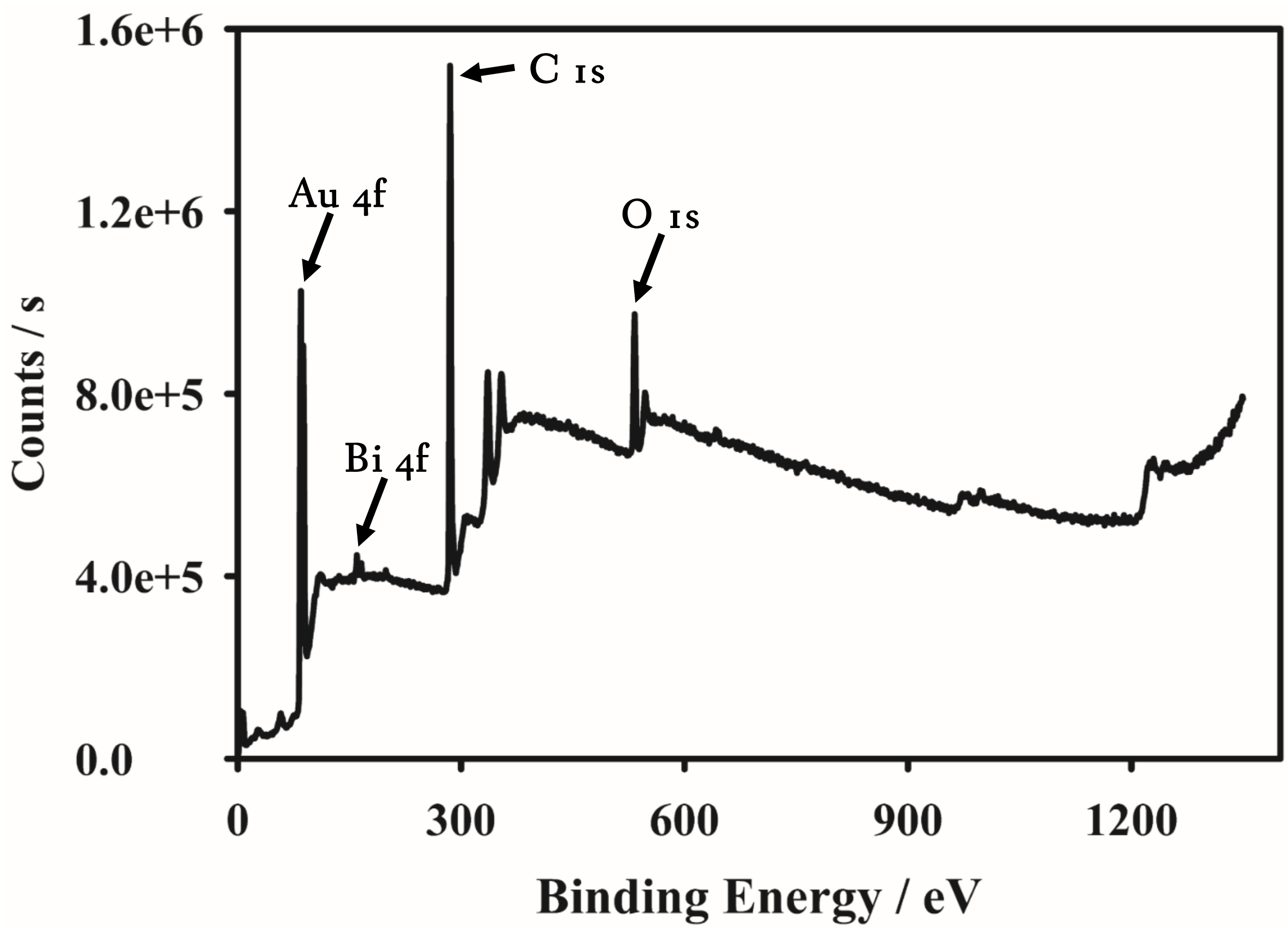

Figure S2. XPS survey spectra of 20 c Au-Bi@GP electrode after electrodeposition and electrochemical testing, showing the characteristic signal of Au, Bi, C, and O with no detectable Pt signal.

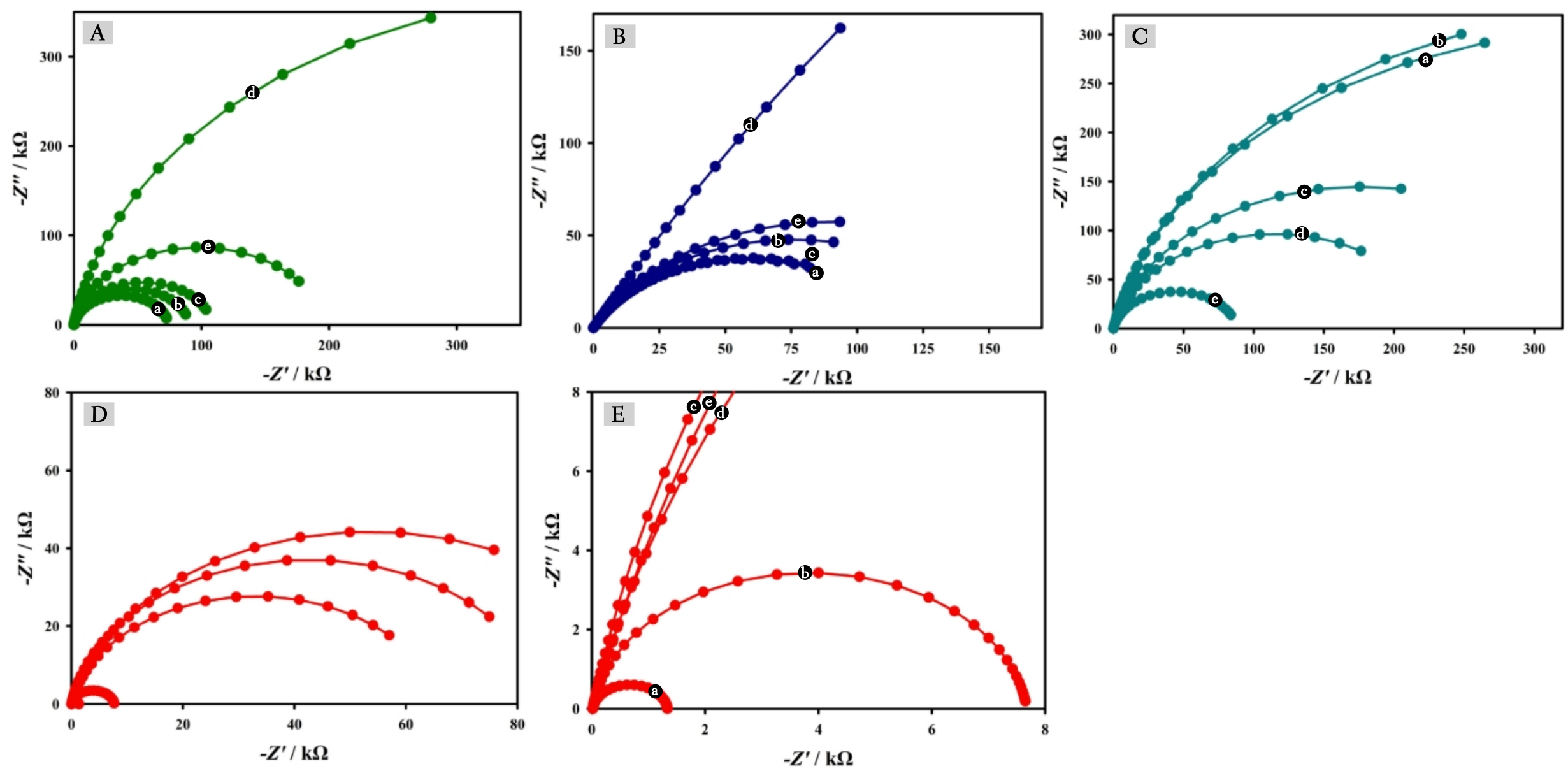

Figure S3. Nyquist plots of the EIS spectra of (A) Bare GP, (B) Au@GP, (C) Bi@GP, (D) Au-Bi@GP, and (E) magnified view of Au-Bi@GP, recorded in 0.5 M H_2SO_4 at (a) −0.297, (b) −0.247, (c) −0.197, (d) 0.097, and (e) 0.003 V vs. RHE.

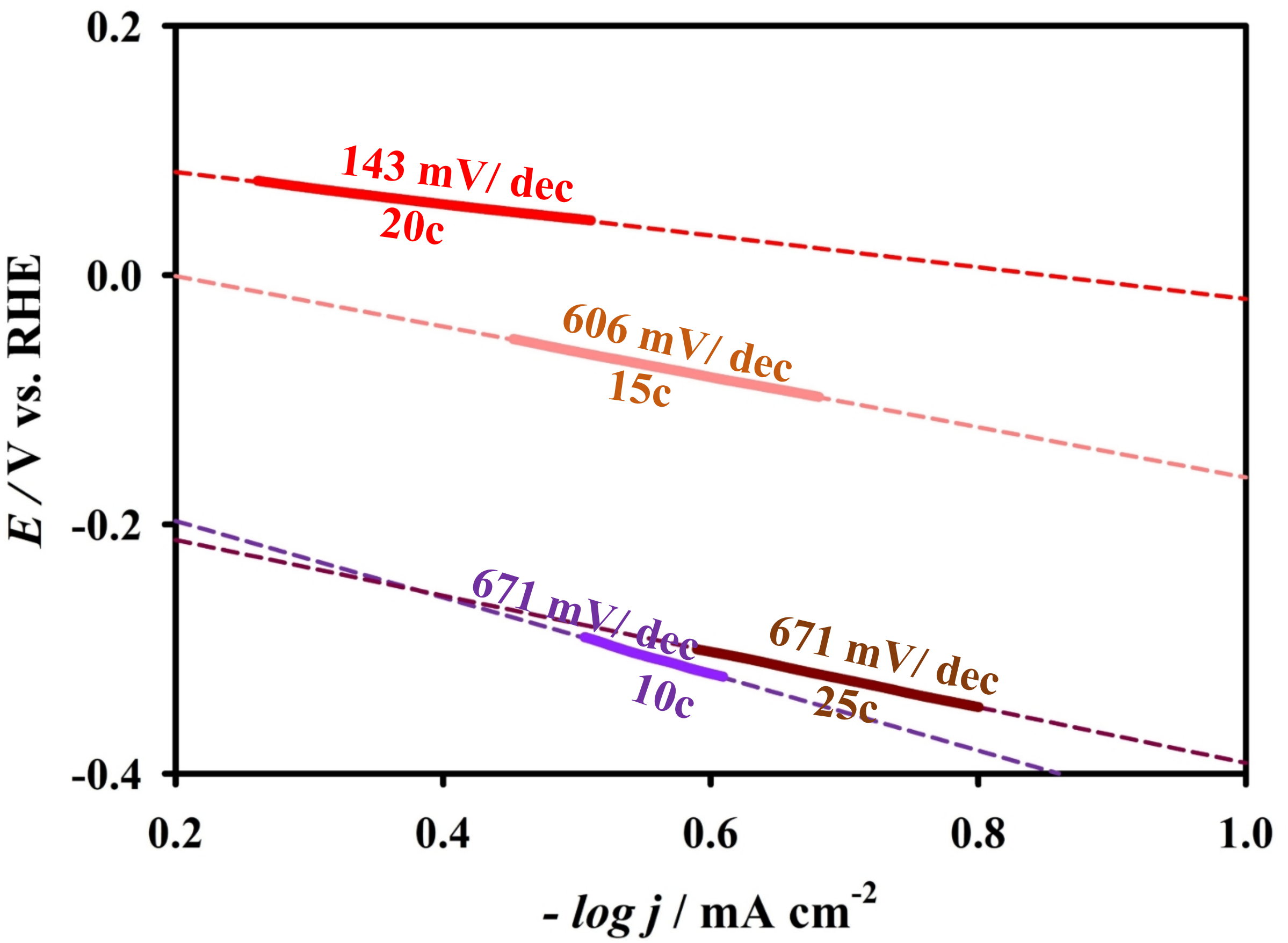

Figure S4. Tafel slope of cycle optimization HER performance of 10, 15, 20 and 25 cycle Au-Bi@GP electrodes.

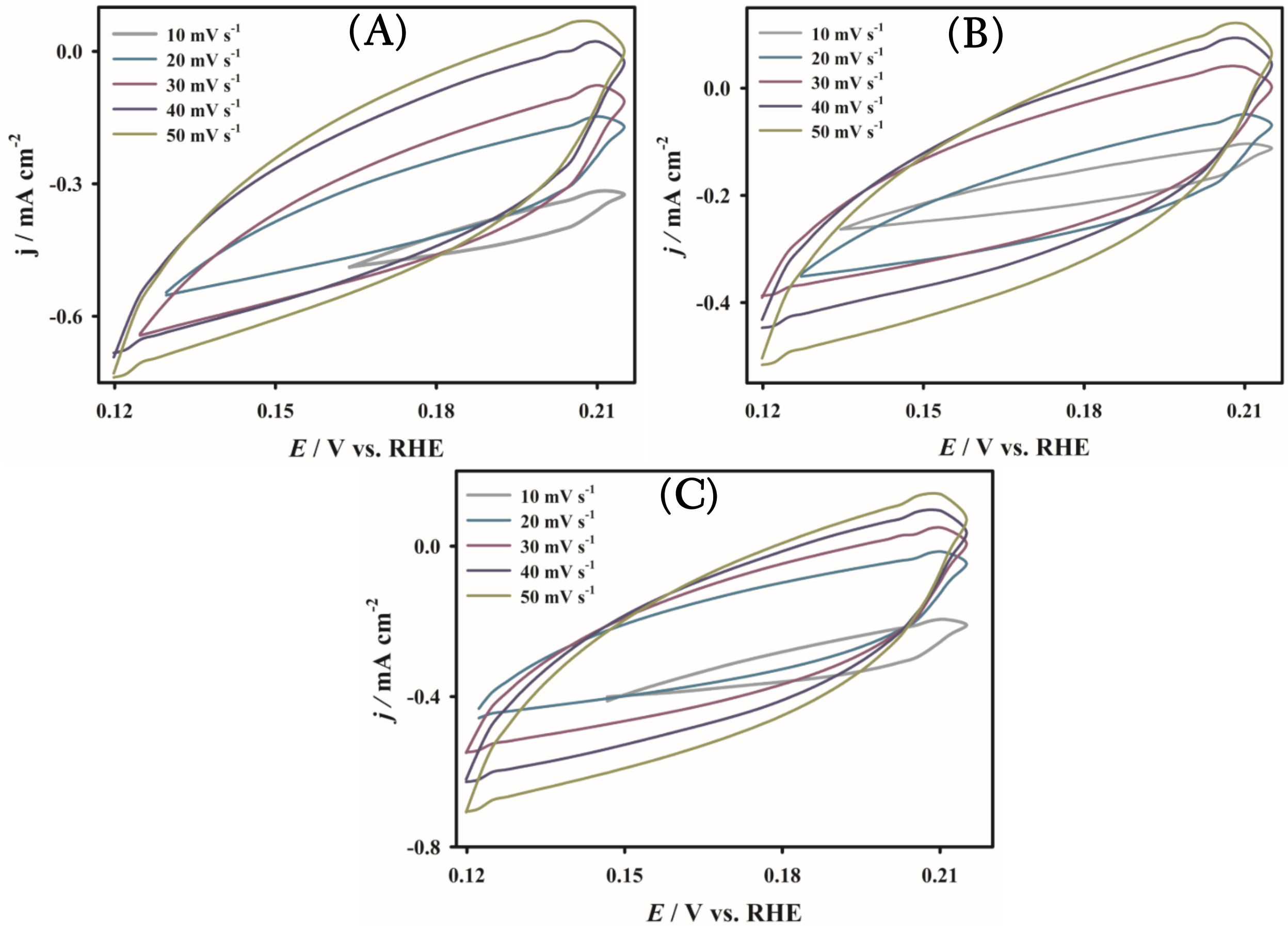

Figure S5. Cyclic voltammograms of (A) Au@GP, (B) Bi@GP, and (C) Au-Bi@GP recorded at scan rates ranging from 10 to 50 mV s^{-1} for the determination of double-layer capacitance C_{dl} and estimation of ECSA.

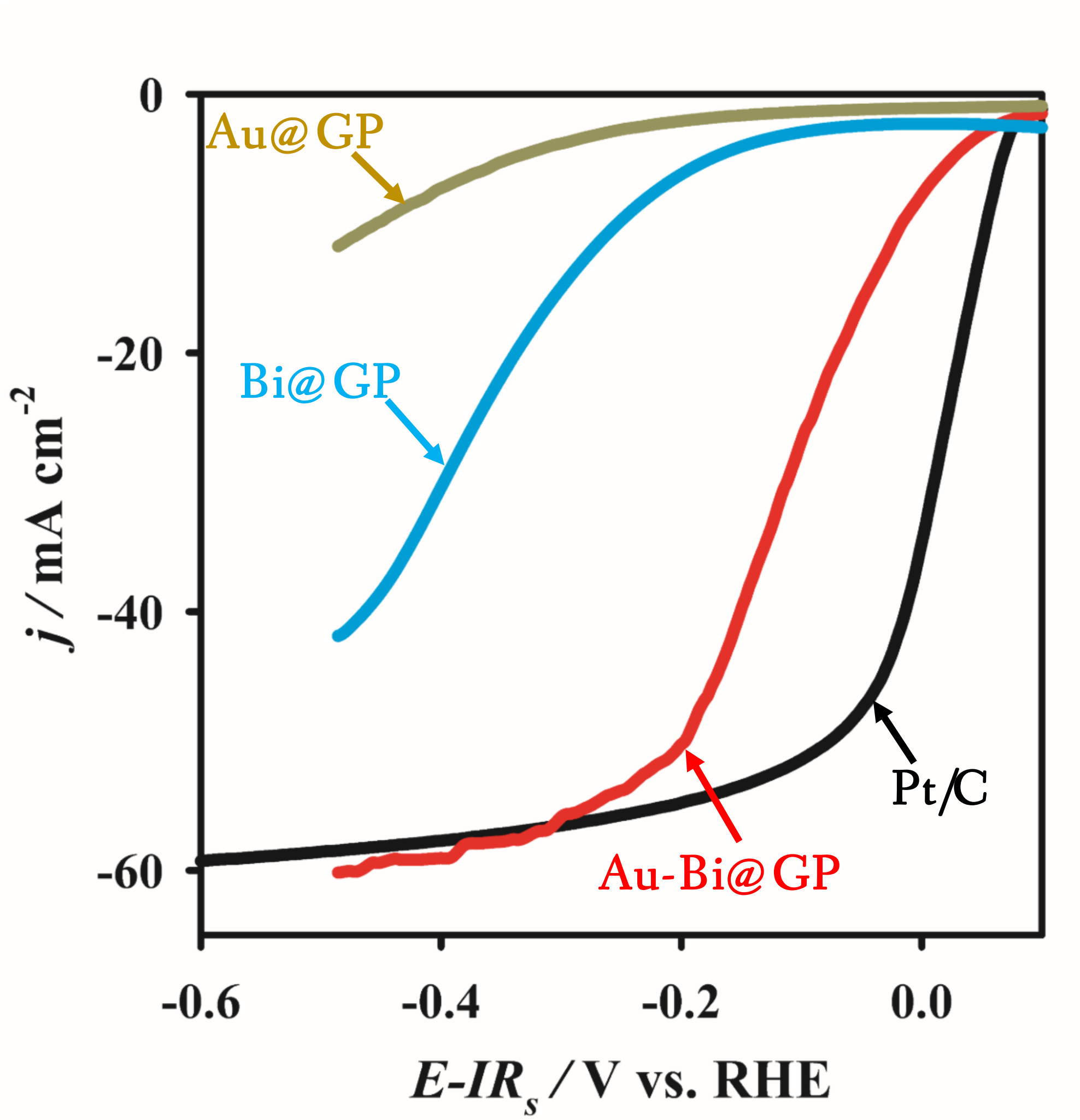

Figure S6. ECSA normalized LSV curves of Au@GP, Bi@GP, Pt/C, and Au-Bi@GP electrodes for HER.

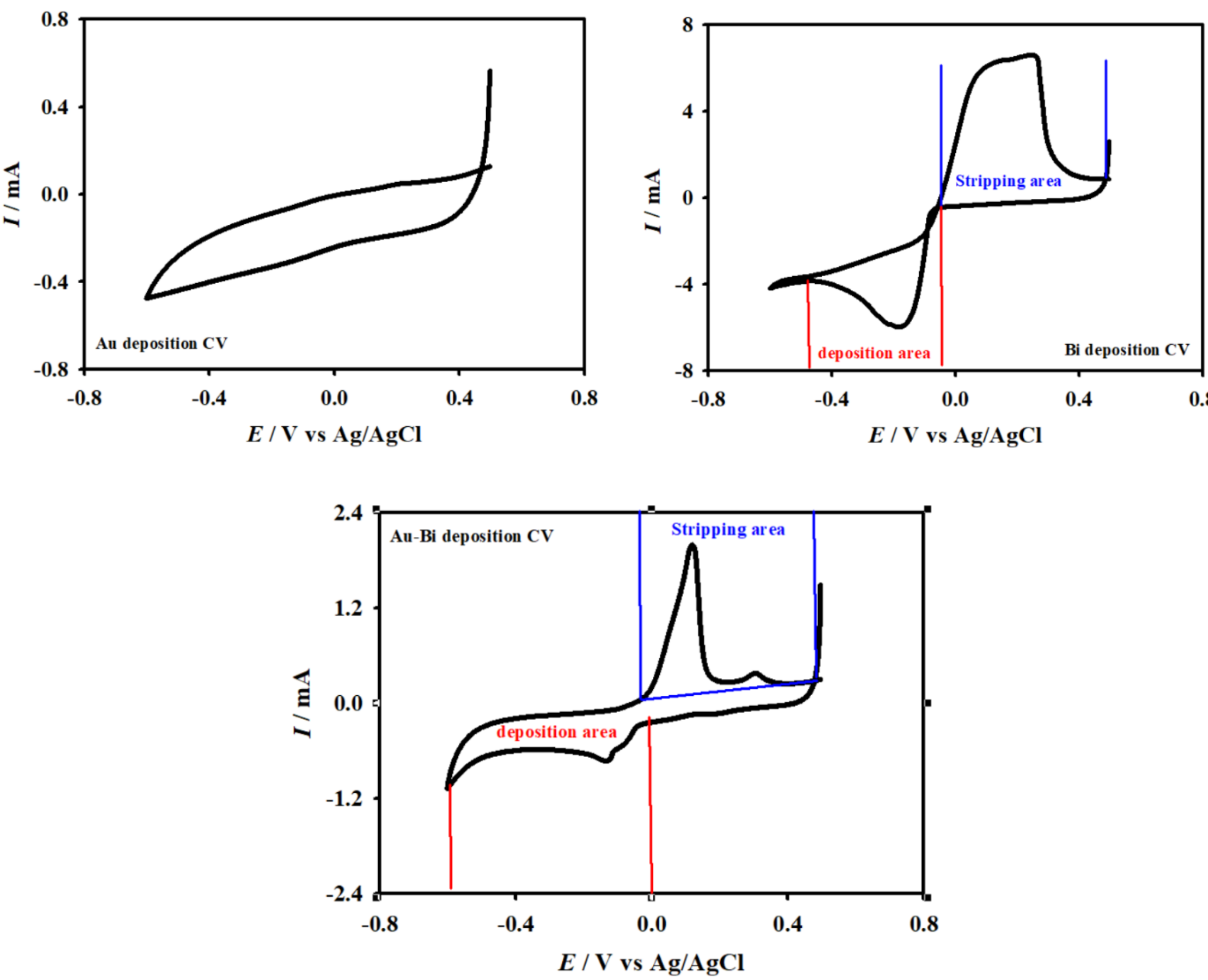

Figure S7. Deposition and stripping voltammograms of Au, Bi, and Au–Bi on the GP electrode.
